# Integration of scRNA-Seq and bulk RNA-Seq uncover perturbed immune cell types and pathways of Kawasaki disease

**DOI:** 10.3389/fimmu.2023.1259353

**Published:** 2023-09-28

**Authors:** Naixin Cao, Huayi Ouyang, Xing Zhang, Yuanyuan Xu, Jun Li, Yanfei Chen

**Affiliations:** ^1^ Yunnan Province Clinical Research Center for Children’s Health and Disease, Kunming Children’s Hospital, Kunming, China; ^2^ Department of Immunology, Center of Immunomolecular Engineering, Innovation and Practice Base for Graduate Students Education, Zunyi Medical University, Zunyi, China

**Keywords:** KD, immune dysregulation, ScRNA-seq, WGCNA, immunopathogenesis

## Abstract

**Introduction:**

Kawasaki disease (KD) is an acute febrile illness primarily affecting children and characterized by systemic inflammation and vasculitis that can lead to coronary artery complications. The aim of this study was to gain a comprehensive understanding of immune dysregulation in KD.

**Methods:**

To this end, we employed integration of single-cell RNA sequencing (scRNA-Seq) and bulk RNA sequencing (bulk RNA-Seq) data. Furthermore, we conducted flow cytometry analysis for a cohort of 82 KD patients.

**Results:**

Our analysis revealed significant heterogeneity within immune cell populations in KD patients, with distinct clusters of T cells, B cells, and natural killer (NK) cells. Importantly, CD4^+^ naïve T cells in KD patients were found to predominantly differentiate into Treg cells and Th2 cells, potentially playing a role in the excessive inflammation and vascular damage characteristic of the disease. Dysregulated signaling pathways were also identified, including the mTOR signaling pathway, cardiomyopathy pathway, COVID-19 signaling pathway, and pathways involved in bacterial or viral infection.

**Discussion:**

These findings provide insights into the immunopathogenesis of KD, emphasizing the importance of immune cell dysregulation and dysregulated signaling pathways. Integration of scRNA-Seq and bulk RNA-Seq data offers a comprehensive view of the molecular and cellular alterations in KD and highlights potential therapeutic targets for further investigation. Validation and functional studies are warranted to elucidate the roles of the identified immune cell types and pathways in KD pathogenesis and to develop targeted interventions to improve patient outcomes.

## Introduction

Kawasaki disease (KD), first identified by Dr. Tomisaku Kawasaki in the late 1960s (1), is an acute febrile illness primarily affecting children, primarily those under the age of five. KD is characterized by systemic inflammation, vasculitis, and the potential for coronary artery aneurysms, making it a significant cause of acquired heart disease in children (2, 3). While KD is considered a rare disease, its incidence has been steadily increasing worldwide and it is thus an area of growing concern for healthcare professionals and researchers. Intravenous immunoglobulin (IVIG) with aspirin is approved as the first-line therapy for KD, including both the initial and refractory stages (4). However, favorable outcomes are not achieved in approximately 10-15% of KD cases (5, 6). Elucidating the etiology, identifying potential risk factors, developing effective treatment strategies, and ultimately reducing the long-term are crucial for children with KD.

Despite several decades of research, the exact cause of KD remains unknown. However, the occurrence of disease in epidemiological clusters (7), environmental conditions (8), and a very low risk of recurrence (9) suggest that KD is triggered by an infectious agent. Development and progression of KD is a concerted effect of multifaceted contributions, mainly including genetic susceptibility, infectious triggers and immunological factors (10). Aberrations in both the innate and adaptive immune responses have been observed in KD, leading to systemic inflammation and vasculitis (11). The numbers of neutrophils, monocytes, and activated γδ T cells in the peripheral blood increases in the acute phase, indicating an enhanced innate immune response in KD patients (12). Moreover, increases in the population of activated T cells along with elevated levels of pro-inflammatory cytokines has been documented in KD patients (13–15). However, no specific pathogen has been associated with KD thus far. By investigating alterations in immune cell subpopulations and signaling pathways, researchers can gain insights into the underlying mechanisms driving the disease, identify potential diagnostic markers, and develop targeted therapies.

Single-cell RNA sequencing (scRNA-seq) enables researchers to identify and analyze distinct cellular subpopulations within disease samples, thereby facilitating a comprehensive understanding of the cellular-level characteristics and mechanisms underlying the disease (16, 17). scRNA-seq has been extensively utilized to reveal the individual differences and diversity of peripheral blood immune cells. Alterations in subpopulations of immune cells and increased levels of cytokines were observed in children with KD, which may lead to aberrant peripheral immune activities (18–20). Analysis of key pathways and candidate genes based on the immune heterogeneity may provide potential immunotherapy targets and meaningful risk prediction for KD. In this study, we analyzed the proportions of peripheral blood immune cells in 82 KD patients, and utilized bulk RNA-seq data and scRNA-seq data to explore the underlying cause of KD.

## Materials and methods

### Peripheral blood collection

A total of 82 peripheral blood samples were collected from children diagnosed with KD at Kunming Children’s Hospital from January 2021 to August 2022. Prior ethical approval was obtained, and informed consent was obtained from the participants and their parents or guardians. The study protocol obtained appropriate ethical committee approval from Kunming Children’s Hospital (2022-03-089-K01). The KD patients conformed to the 2017 American Heart Association (AHA) KD diagnostic guidelines and were not treated with any drugs prior to hospitalization. The clinical data and serological test results of the children diagnosed with KD were acquired from Kunming Children’s Hospital, and the statistical results are showed in **Supplementary Table 1**. Blood collection was performed during the early phase, typically 5 to 10 days after the onset of fever. Trained health care personnel followed aseptic techniques to collect approximately 2 milliliters of peripheral blood from each participant. Sample collection was followed by flow cytometry detection.

### Flow cytometry analysis

Peripheral blood samples, collected and anticoagulated with EDTA, were obtained and processed within 48 hours at ambient room temperature. A volume of 50 µL of peripheral blood was added to conventional flow cytometry sample tubes. Subsequently, 20 µL of BD MultitestTM 6C-TBNK reagent (catalog number 644611) comprising the following monoclonal antibodies was added to each sample: CD3 FITC (for T cell identification), CD16 & CD56 PE (for NK cell identification), CD45 PerCP-Cy5.5 (for lymphocyte subtyping), CD4 PE-Cy7 (for T helper cell identification), CD19 APC (for B cell identification), and CD8 APC-Cy7 (for cytotoxic T cell subset identification). Gentle vortexing ensured thorough mixing. The samples were incubated in a light-protected environment at room temperature for 15 minutes. Following incubation, 450 µL of 1× FACS lysis solution was added into each sample, and the mixture was gently vortexed. The samples were again incubated in a light-protected environment at room temperature for an additional 15 minutes. Flow cytometry analysis was conducted within a maximum of six hours after completion of the incubation period. The data derived from the flow cytometry analysis provided precise quantification of the proportions and absolute counts of CD3^+^ T cells, CD4^+^ T cells, CD8^+^ T cells, B cells, and NK cells within the peripheral blood samples.

### Data acquisition and processing

The KD-related datasets utilized in this study [GSE68004 (21) and GSE168732 (18)] were downloaded from the NCBI Gene Expression Omnibus (GEO; https://www.ncbi.nlm.nih.gov/geo/) database. The GSE68004 series was employed for WGCNA and assessment of the immune cell landscape, which encompassed 86 KD patients samples (13 incomplete KD and 76 complete KD) and 37 healthy volunteers. Six untreated KD samples used for scRNA-seq analysis were extracted from GSE168732 data.

### Weighted gene co-expression network analysis

Raw transcriptome data were preprocessed using R packages, including quality control assessment, normalization, and log transformation of the gene expression values to ensure data reliability and comparability. The WGCNA package was utilized to construct a weighted gene co-expression network. This involved calculating pairwise correlations between genes, followed by the transformation of the correlation matrix into an adjacency matrix using a chosen soft thresholding power. The co-expression network was clustered into distinct modules using hierarchical clustering algorithms. Modules are sets of genes that are highly interconnected, indicating potential functional relationships. The relationship between modules and traits of interest (disease, age and gender) was investigated. This involved correlating the module eigengenes with the trait of interest and performing statistical tests to determine the significance of the associations. Subsequently, the relationship between key module and disease status was determined using Pearson correlation analysis. The correlation among gene expression in candidate module was calculated and defined as MM value (the Person correlation coefficient of Module Membership). A gene significance (GS) was defined as mediated p-value of each gene (GS = lgP) in the linear regression between gene expression and the clinical traits. We set as the cut-off criteria (|GS| > 0.2 and |MM| > 0.9) to screen hub genes. These steps were performed using R software (version 4.2.2). The specific R code and parameters used for each step can be provided upon request.

### Differentially expressed genes identification

To identify candidate genes from the key module, differential gene expression analysis was performed in R with the package of DESeq2. The data were preprocessed and normalized using the DESeq function. Differential gene expression was detected by calculating statistics and P-values using the results function.

### Assessment of immune cell landscape

The CIBERSORT (cell-type identification by estimating relative subsets of RNA transcripts) algorithm was applied to estimate the relative proportions of different immune cell types within the transcriptome data (which were preprocessed in the above step). This algorithm utilizes a predefined reference gene expression matrix to infer the abundance of immune cell populations. In total, 22 types of immune cells were evaluated. The proportion and differentially changed immune cells between KD and controls were fully analyzed and visualized. To validate the accuracy of immune cell deconvolution, flow cytometry was performed on a subset of samples, as described previously.

### scRNA-seq data processing

scRNA-seq data for KD were obtained by selecting GSE168732 from the GEO database. A total of 6 untreated KD samples were chosen for subsequent analysis. The obtained dataset containing 37,403 cells was subjected to quality control using the Seurat package (version 4.3.0) in R software (version 4.1.3). Principal component analysis (PCA) was performed using the JackStraw and PCEIbow Plot functions (22). Essential principal components were chosen for subsequent dimensionality reduction and clustering analysis. By utilizing the selected important principal components, dimensionality reduction and clustering analysis were performed using the t-SNE (t-distributed stochastic neighbor embedding) and UMAP (uniform manifold approximation and projection) algorithms. The t-SNE and UMAP methods were employed to map high-dimensional data onto two- or three-dimensional space, enabling visualization and differentiation of cell clusters. Cell type annotation of the clusters was conducted using the singleR package (version 1.6.1) and the Monaco algorithm. SingleR was used to match individual cell RNA-seq data with known reference datasets to determine the cell types within each cluster. Manual correction was performed using CellMarker (23) to enhance the accuracy and reliability of cell type annotation. To further identify cell types in detail, UMAP dimensionality reduction and clustering analysis were re-performed on individual cell clusters. This step aided in the finer resolution and identification of more specific cell subtypes.

### Statistical analysis

All statistical analyses were performed using R software (version 4.2.2) and GraphPad Prism (version 5.01). Student’s t-test was used to compare values between the test and control groups and P-values < 0.05 were considered significant.

## Results

### Identification of key modules and candidate genes by WGCNA

**Figure 1:**
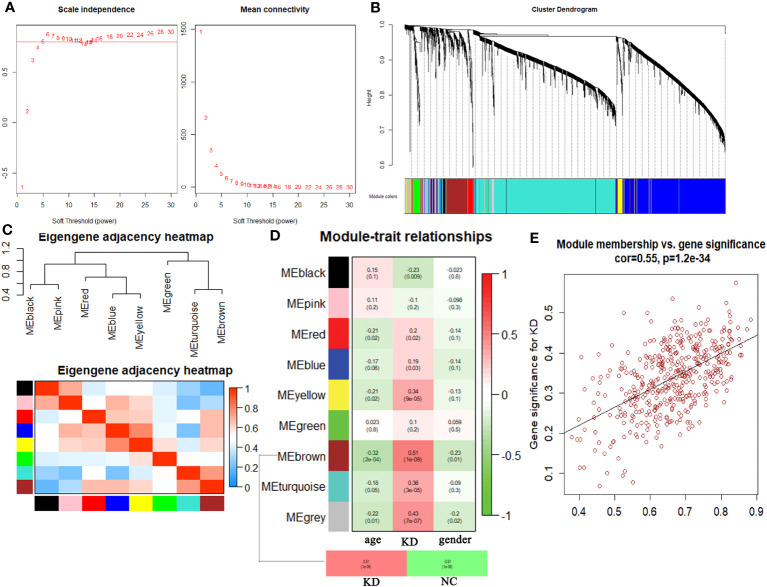
Comprehensive weighted gene co-expression network analysis. **(A)** Selection of soft threshold power value. **(B)** Clustering dendrogram of all genes based on dissimilarity algorithm and assignment modules. **(C)** Cluster dendrogram and heatmap of adjacency eigenvalue in the network. **(D)** Module-trait correlation heatmap between clinical traits and modules. **(E)** Correlation scatter plot between gene significance and module membership in brown module.

Hierarchical clustering analysis detected heterogeneity within each sample, and a total of 126 samples and 4827 genes were included for subsequent research. Subsequently, WGCNA was conducted, leading to the identification of co-expression modules. The determination of the soft threshold power β was achieved at 6 when the R^2^ value reached 0.90, and the mean connectivity approached 0 (**Figure 1A**). A total of eight modules were obtained by hierarchical clustering tree analysis (**Figure 1B**). Notably, the brown module exhibited a strong correlation with the turquoise module, and the blue module displayed a high correlation with the yellow module (**Figure 1C**). Further analysis was carried out to examine the relationship between these modules and clinical features (**Figure 1D**). Remarkably, the brown module demonstrated the highest correlation with KD (P<0.01). To gain deeper insights into the brown module and the overall variation trend of genes in KD, scatter plots were generated to depict the relationship between module membership and gene significance. The results indicated a positive correlation between the brown module and the disease state (**Figure 1E**).

**Figure 2:**
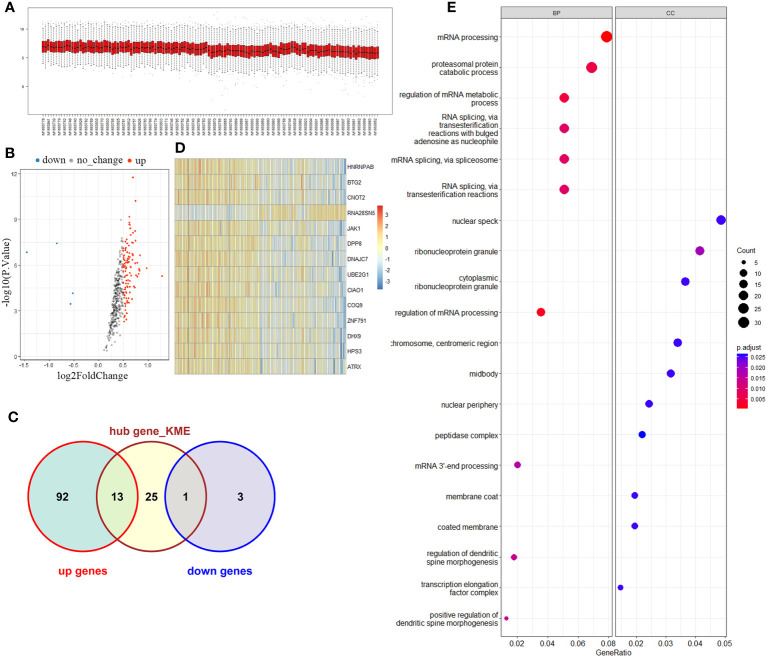
Key module and candidate genes analysis. **(A)** Brown module genes expression among normal and KD groups. **(B)** Volcano map of differentially expressed genes in the brown module. **(C)** Venn diagram of differentially expressed genes and Hub genes. **(D)** Overlapping genes expression heatmap. **(E)** Functional enrichment analysis of overlapping genes.

The brown module comprised a total of 421 genes. Upon testing the expression levels of these genes in all samples, it was observed that they exhibited a relatively concentrated distribution, indicating that the gene expression levels within this module were consistently within the same range (**Figure 2A**). The correlation among gene expression in the brown module was calculated and defined as MM value (the Person correlation coefficient of Module Membership). A gene significance (GS) was defined as mediated p-value of each gene (GS = lgP) in the linear regression between gene expression and the clinical traits. We set as the cut-off criteria (|GS| > 0.2 and |MM| > 0.9) to screen hub genes, and 39 hub genes were found. Simultaneously, differential analysis of the genes in the brown module was performed, and a total of 109 differentially expressed genes (DEG) were identified. Among the DEGs, 105 were upregulated and 4 downregulated (**Figure 2B**). Notably, 14 genes overlapped between the set of DEGs and the hub gene set selected from the brown module (**Figure 2C**). These genes included *HNRNPAB*, *BTG2*, *CNOT2*, *RNA28SN5*, *JAK1*, *DPP8*, *DNAJC7*, *UBE2G1*, *CIAO1*, *COQ9*, *ZNF791*, *DHX9*, *HPS3*, and *ATRX*. To further assess the discriminatory ability between the control group and KD group, a heatmap was generated to visualize the expression patterns of these 14 overlapping genes. The results demonstrated a strong discriminatory ability between the two groups (**Figure 2D**). Moreover, GO analysis was performed to explore the functions of these candidate genes, and results suggested that patients with Kawasaki disease may experience significant proliferation of peripheral blood cells (**Figure 2E**).

### Immune landscape in KD

**Figure 3:**
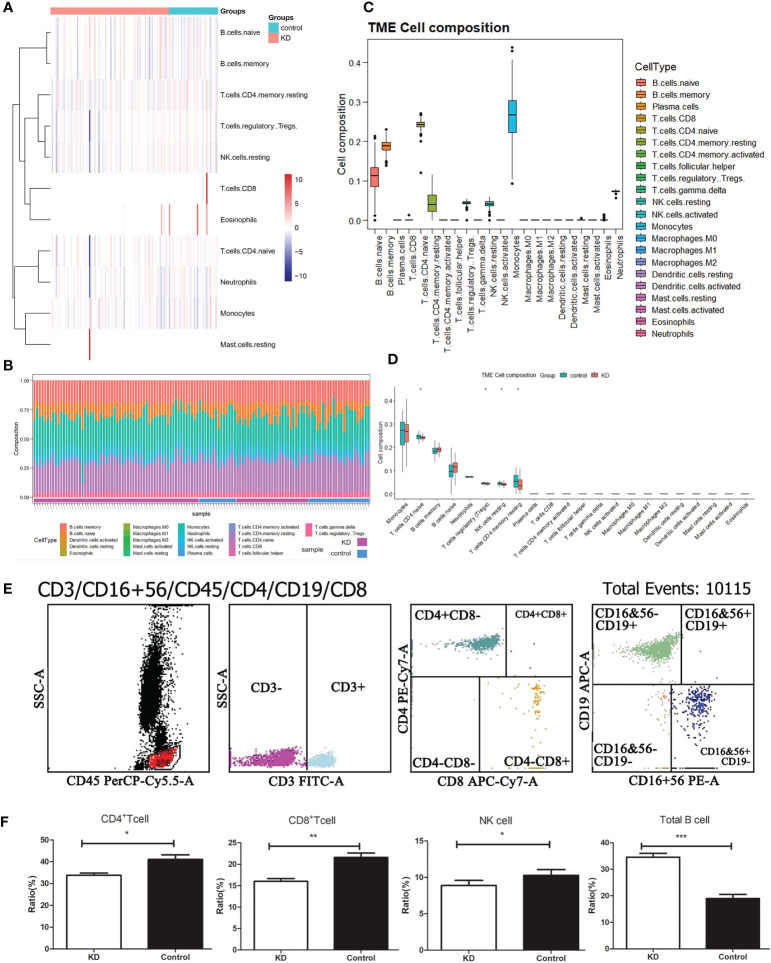
Immune landscape analysis. **(A)** Heatmap visualizing the distribution of 22 types of immune cells from normal and KD patients. **(B)** Box plot showing the whole composition of immune cells. **(C)** Pile-up histogram displaying proportion of immune cells in each sample. **(D)** Differentially analysis of immune cells between normal and KD patients. **(E)** The proportion of peripheral blood immune cells in children with KD by flow cytometry. **(F)** Differentially analysis of immune cells between normal and KD patients based on flow cytometry results. *P < 0.05; **P < 0.01; ***P < 0.001.

Despite limited understanding of the pathogenesis of KD, several epidemiologic phenomena suggest the involvement of an unidentified infectious agent that triggers immune system activation. To delve deeper into the distinct immune landscape between KD patients and healthy volunteers, we conducted analysis of immune landscape by utilizing bulk RNA sequencing data. **Figures 3A, B** present the distribution and detailed proportions of 22 immune cell types in all participants. Notably, monocytes and CD4^+^ naïve T cells constituted the major components in the peripheral blood of both KD patients and healthy volunteers (**Figure 3C**). Furthermore, significant alterations in cell composition were observed in KD. CD4^+^ naïve T cells, Tregs, NK cells, and CD4^+^ memory T cells exhibiting notable differences (P<0.05) (**Figure 3D**).

To validate these alterations in the immune cell landscape in KD, we employed flow cytometry to examine the levels of total T cells, B cells, CD4^+^ T cells, CD8^+^ T cells, and NK cells in the peripheral blood of 82 KD patients (**Figure 3E**). Our findings revealed that CD4^+^ T cells, CD8^+^ T cells, and NK cells were significantly decreased in KD patients compared to the control group, whereas the proportion of B cells was significantly elevated in KD patients (**Figure 3F**). Despite the decrease in both CD4^+^ and CD8^+^ T cells in KD, the ratio of CD4^+^/CD8^+^ T cells was higher in the KD group than in the control group.

### Immune cellular heterogeneity in KD by scRNA-seq

**Figure 4:**
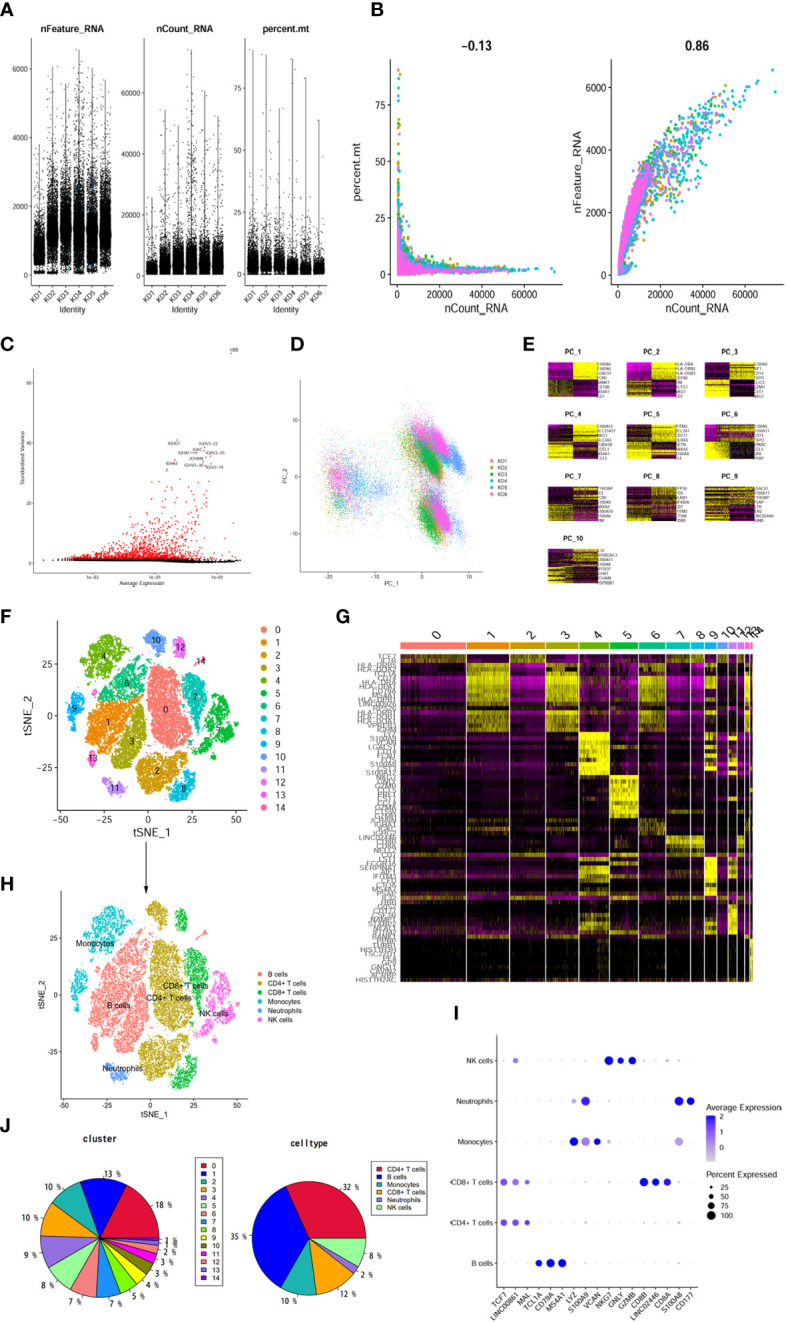
The dimension reduction of KD scRNA-seq. **(A)** After standard quality control of all cells from 6 patients, 37403 cells were included in the analysis. **(B)** The numbers of detected genes were significantly related to the sequencing depth, with a high Pearson’s correlation coefficient 0.86; the numbers of detected mitochondria were the same among different sequencing depth. **(C)** Screen out the top 1500 characteristic genes with the highest expression difference. Red dots represented highly variable genes and black dots represented non-variable genes. **(D)** PCA scatter plot displayed dots distribution after integration analysis. **(E)** Use the DimHeatmap function to display gene expression heatmaps of the top 10 principal components, the top 8 principal components showed significant differences. **(F)** tSNE dimensional reduction method was applied with the top 8 PCs, and 15 cell clusters were classified. **(G)** The top 5 marker genes for each cluster were displayed in heatmap. **(H)** tSNE plot after cell type annotation for each cluster. **(I)** The relationship between the expression level of marker genes and cell populations. **(J)** Proportion of each cluster and cell type.

To gain insights into the transcriptomic landscape at the single-cell level, scRNA-seq data from six KD samples were obtained from the GEO dataset. A total of 37,403 cells were initially included in the analysis. By following rigorous quality control measures (**Figures 4A, B**), 31,485 cells were identified as meeting the criteria. Subsequently, variance analysis identified 1,500 highly variable genes (**Figure 4C**). PCA dimensionality reduction analysis demonstrated substantial overlap among cells from KD patients (**Figure 4D**), indicating successful integration. Further principal component analysis was performed, and the top 8 principal components with the highest P-values were selected for subsequent analysis (**Figure 4E**). Clustering analysis using the tSNE algorithm revealed 15 distinct cell clusters (**Figure 4F**), demonstrating considerable heterogeneity among different cell populations (**Figure 4G**). The 15 clusters were then annotated into 6 cell types (**Figure 4H**), which included B cells, CD4^+^ T cells, CD8^+^ T cells, monocytes, NK cells, and neutrophils. CD4^+^ T cells encompassed clusters 0, 3, 8, and 11, as characterized by specific marker genes such as *TCF7*, *LINC00861*, and *MAL*. Monocytes were represented by clusters 4 and 10, with marker genes *LYZ*, *S100A9*, and *VCAN*. NK cells were identified in clusters 5 and 10, expressing marker genes *NKG7*, *GNLY*, and *GZMB*. CD8+ T cells were found in clusters 7 and 9, as marked by genes *CD8B*, *LINCO2446*, and *CD8A*. Neutrophils were assigned to cluster 12, with marker genes *S100A8* and *CD177*. B cells were represented by clusters 1, 2, 6, 13, and 14, as indicated by high expression of marker genes *TCL1A*, *CD79A*, *MS4A1*, among others (**Figure 4I**). Comparison of cell population proportions before and after annotation revealed that both CD4^+^ T cells and B cells accounted for over 30% of the total cell population (**Figure 4J**).

**Figure 5:**
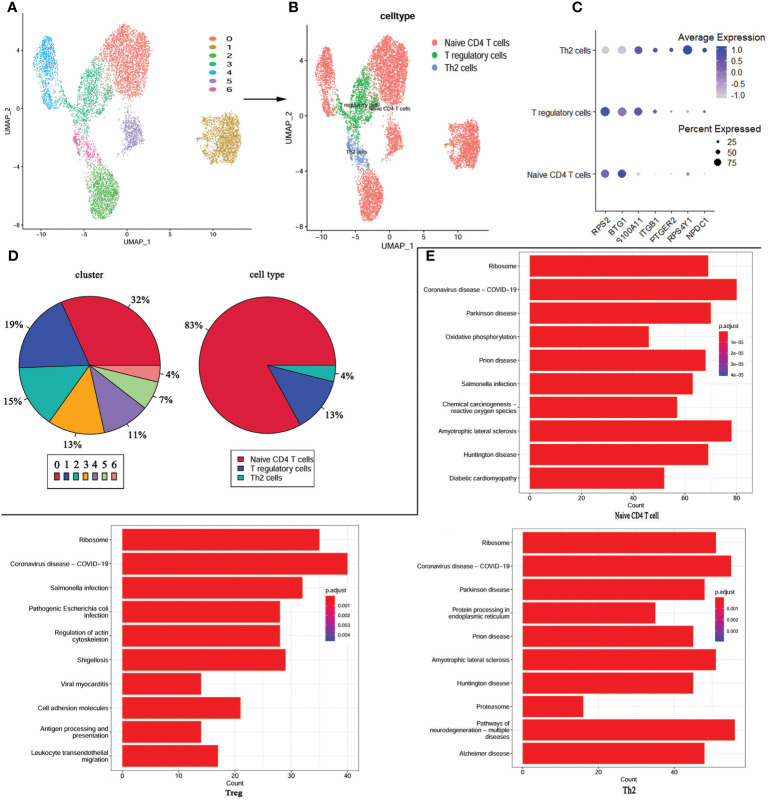
re-UMAP analysis of CD4^+^T cells extracted from whole cells. **(A)** UMAP plot of CD4^+^ T cell subpopulation. **(B)** UMAP plot after cell type annotation for each cluster. **(C)** The relationship between the expression level of marker genes and cell populations. **(D)** Proportion of each cluster and cell type. **(E)** Top 10 pathways enriched in the three cell groups.

As these changes in the immune landscape suggest a high correlation between CD4^+^T cells and KD, so we extracted CD4^+^ T cells from the scRNA-seq data and performed re-analysis using UMAP. Consequently, the CD4^+^ T cells were re-clustered into 7 distinct clusters (**Figure 5A**). According to the expression of marker genes, CD4+ T cells were classified into Tregs, Th2 cells, and CD4+ naïve T cells using singleR and cellmarker algorithms (**Figure 5B**). Differential expression analysis of the associated core markers (*RPS2*, *BTG1*, *S100A11*, *ITGB1*, *PTGR2*, *RPS4Y1*, and *NPDC1*) in the cells was shown in **Figure 5C**. The pie chart represents the proportion of each cell population before and after annotation, revealing that CD4^+^ naïve T cells accounted for 83% of the population, followed by Tregs at 13%, and Th2 cells at 4% (**Figure 5D**).

For a more in-depth study of the detailed cellular trajectory of CD4^+^ T cells, we use the differentially expressed genes in the CD4^+^ T cell group’s clusters to mark the cells (**Supplementary Figure 1**). After dimension reduction, the marked cells were sorted according to the trajectory, resulting in one root part and two branches. Regulatory T cells and Th2 cells were located in branch 1 and branch 2 at the beginning of node 1, while the CD4^+^ naïve T cells were mostly located at the root, and some were within the range between branch 1 and branch 2 and node 1. Subsequently, KEGG was performed based on the three cell types. The COVID-19 signaling pathway was significantly enriched in all three types of cells, and the signaling pathways co-enriched in Treg and Th2 cells were mostly associated with bacterial or viral infections and cardiomyopathy (**Figure 5E**).

## Discussion

Kawasaki disease is a complex and enigmatic pediatric illness characterized by systemic inflammation and vasculitis, often leading to coronary artery complications. In this study, we sought to gain a deeper understanding of the immune dysregulation that underlies KD by employing a multi-faceted approach, integrating single-cell RNA sequencing data, bulk RNA sequencing data, and flow cytometry analysis in a cohort of 82 KD patients. Our results provide valuable insights into the immunopathogenesis of KD.

Kawasaki disease exhibits intricate immune cell dysregulation, encompassing a range of immune cell types (24–26). Our scRNA-Seq analysis and detection of peripheral blood immune cells in patients with Kawasaki disease by flow cytometry revealed substantial heterogeneity within their immune cell populations, emphasizing the complex nature of the immune response in KD. We identified distinct clusters of immune cells, including T cells, B cells, and natural killer cells. Furthermore, we observed significant differences in the distribution and activation states of these immune cell subsets compared to healthy controls. These findings support that KD is characterized by a dysregulated immune response involving multiple immune cell types (9, 27).

CD4^+^ T cells play a crucial role in the immune response and are involved in Kawasaki disease (28, 29). Several studies have shown alterations in the subset distribution and function of CD4^+^ T cells in Kawasaki disease patients (30, 31). In this study, CD4^+^ T cells were a focal point in single-cell sequencing, flow cytometry analysis, and bulk RNA-seq analysis. Therefore, we performed re-clustering of CD4^+^ T cells from Kawasaki disease patients, including the CD4^+^ naïve T cells, Th2 cells, and Treg cells. Th2 cells are responsible for regulating antibody production and promoting immune responses against extracellular pathogens (32). In Kawasaki disease, there is evidence of imbalanced Th1/Th2 cell responses, and the plasma level and mRNA expression levels of Th1 cytokines (*IFN-γ*, *IL-2*) and Th2 cytokines (*IL-4* and *IL-10*) are markedly elevated during the acute stage of Kawasaki disease (33, 34). This imbalance may contribute to the abnormal immune response observed in the disease (increased inflammatory response). Tregs are involved in immune regulation and suppressing excessive immune responses (35). Studies have shown alterations in the number and function of Tregs in Kawasaki disease patients, indicating a potential role in the dysregulated immune response (36–38). The specific contribution of CD4^+^ T cell subsets in Kawasaki disease is still being investigated, and further research is needed to understand their exact roles in disease pathogenesis. The dysregulation of CD4^+^ T cell subsets in Kawasaki disease suggests an imbalance in the immune response, which may contribute to the excessive inflammation and vascular damage observed in the disease.

It is noteworthy that our results provide further evidence linking KD with COVID-19, as all three CD4^+^ T cell clusters from KD patients were found to cluster within the COVID-19 signaling pathway. Several studies have reported an increase in cases of Kawasaki disease during the COVID-19 pandemic (39, 40). These cases often have overlapping symptoms with those of COVID-19, such as fever and inflammation (41). The specific relationship between Kawasaki disease and COVID-19 is not yet fully understood. In both Kawasaki disease and severe COVID-19 cases, an excessive immune response can lead to widespread inflammation known as a cytokine storm (42). The dysregulation of these cytokines can lead to the activation of various cellular processes, increasing inflammation and potentially causing damage to blood vessels. Further research is needed to understand the exact mechanisms underlying the relationship between Kawasaki disease and COVID-19. Investigating these signaling pathways may not only provide insights into the development of Kawasaki disease in COVID-19 patients but also shed light on potential therapeutic targets for both conditions.

In recent years, significant progress has been made in unraveling the genetic factors contributing to KD susceptibility and pathogenesis (9, 10, 43). In this study, we identified several key genes associated with KD, such as *HNRNPAB*, *BTG2*, *CNOT2*, *RNA28SN5*, *JAK1*, *DPP8*, *DNAJC7*, *UBE2G1*, *CIAO1*, *COQ9*, *ZNF791*, *DHX9*, *HPS3*, and *ATRX*. Among them, *JAK1* is a key mediator in the Janus kinase (JAK)-signal transducer and activator of transcription (STAT) signaling pathway, which is critical for immune responses (44), potentially contributing to excessive inflammation that occurs in KD. Moreover, we identified several key pathways that were significantly perturbed in KD patients. Although numerous studies suggest that Kawasaki disease is associated with infection, no specific pathogenic agent has been confirmed yet (45). Several signaling pathways related to bacterial and viral infections were discovered in this study, indicating that the clinical symptoms of KD may be caused by activation of central links in the immune response process, without specific antigen stimulation. This may explain the correlation between Kawasaki disease and autoimmune diseases (46, 47) and superantigens (48, 49). Additionally, pathways involved in cardiomyopathy exhibited dysregulation, suggesting their contribution to the development of coronary artery complications and heart disease in KD.

Integration of scRNA-Seq and bulk RNA-Seq data provided a comprehensive view of the immune dysregulation in KD, allowing us to bridge the gap between the cellular and molecular levels. This integrative approach revealed novel candidate genes and regulatory networks that may be involved in KD pathogenesis. These findings open up avenues for future research aimed at validating the functional roles of these genes and exploring their potential as therapeutic targets. However, it is important to acknowledge the limitations of our study. The sample size of our cohort was relatively small, and further studies with larger cohorts are warranted to validate our findings and identify potential subgroups within KD. Additionally, the cross-sectional nature of our study limited our ability to determine causal relationships between the identified immune cell perturbations, signaling pathways, and disease progression.

In conclusion, our study provides comprehensive insights into the perturbed immune cell types and signaling pathways associated with Kawasaki disease. The integration of scRNA-Seq and bulk RNA-Seq data allowed us to uncover the heterogeneity within immune cell populations and identify dysregulated pathways involved in inflammation and cardiomyopathy. These findings contribute to a better understanding of the immunopathogenesis of KD and provide potential targets for therapeutic interventions. Further research is needed to validate these findings and explore the functional significance of the identified immune cell subpopulations and signaling pathways in the context of KD.

## Data availability statement

The original contributions presented in the study are included in the article/**Supplementary Material**. Further inquiries can be directed to the corresponding authors.

## Ethics statement

The studies involving humans were approved by Medical Ethics Committee of Kunming Children’s Hospital. The studies were conducted in accordance with the local legislation and institutional requirements. Written informed consent for participation in this study was provided by the participants’ legal guardians/next of kin.

## Author contributions

JL: Conceptualization, Supervision, Writing – original draft, Writing – review & editing. NC: Data curation, Formal Analysis, Resources, Software, Visualization, Writing – original draft. HO: Data curation, Formal Analysis, Methodology, Resources, Software, Visualization, Writing – original draft. XZ: Conceptualization, Data curation, Funding acquisition, Resources, Supervision, Writing – original draft. YX: Data curation, Formal Analysis, Investigation, Validation, Writing – original draft. YC: Conceptualization, Funding acquisition, Supervision, Writing – original draft, Writing – review & editing.
