# Supplementary material for: Integration of scRNA-Seq and bulk RNA-Seq uncover perturbed immune cell types and pathways of Kawasaki disease
Source: Front Immunol. 2023 Sep 28;14:1259353. doi: 10.3389/fimmu.2023.1259353 (PMC10568768; doi:10.3389/fimmu.2023.1259353)
Supplement: Supplementary file 1 [file DataSheet_1.docx]

Supplement Table 1 Statistics of clinical indicators and serological test results of the children diagnosed with kawasaki disease.

| Category | Object | Detection value |
| --- | --- | --- |
| Main clinical manifestations | Fever | 82/0 |
|  | Changes in the oral and labial mucosa | 68/14 |
|  | Bilateral non exudative conjunctival congestion | 70/12 |
|  | Rash | 53/29 |
|  | Changes in limb endings | 32/50 |
|  | Cervical lymph node | 42/40 |
| Routine blood test | Leukocyte (10^9/L) | 13.2±5.5 |
|  | Platelet count (10^9/L) | 469.4±169.9 |
|  | Hemoglobin (g/L) | 111.5±11.1 |
|  | Lymphocyte count (10^9/L) | 4.1±1.9 |
|  | Neutrophil count (10^9/L) | 7.4±4.2 |
|  | Monocytes count (10^9/L) | 0.8±0.4 |
|  | C-reactive protein (mg/L) | 67.1±43.9 |
| Liver and kidney function index | Total bilirubin(umol/L) | 8.1±3.1 |
|  | Albumin (g/L) | 32.3±3.9 |
|  | Glutamic-pyruvic transaminase (U/L) | 38.9±33.2 |
|  | Glutamic oxaloacetic transaminase (U/L) | 29.7±10.7 |
| Lipid index | Total cholesterol (mmol/L) | 3.4±0.8 |
|  | Triglyceride (mmol/L) | 1.4±0.5 |
| Coagulation index | Prothrombin time (s) | 13.9±3.7 |
|  | D-Dimer (mg/L) | 1.8±1.3 |
|  | Activated partial thromboplastin time (s) | 40.1±6.1 |
| Gender | Girl | 31 |
|  | Boy | 51 |
| Clinical diagnosis | Complete KD | 58 |
|  | Incomplete KD | 24 |

Supplement Figure 1


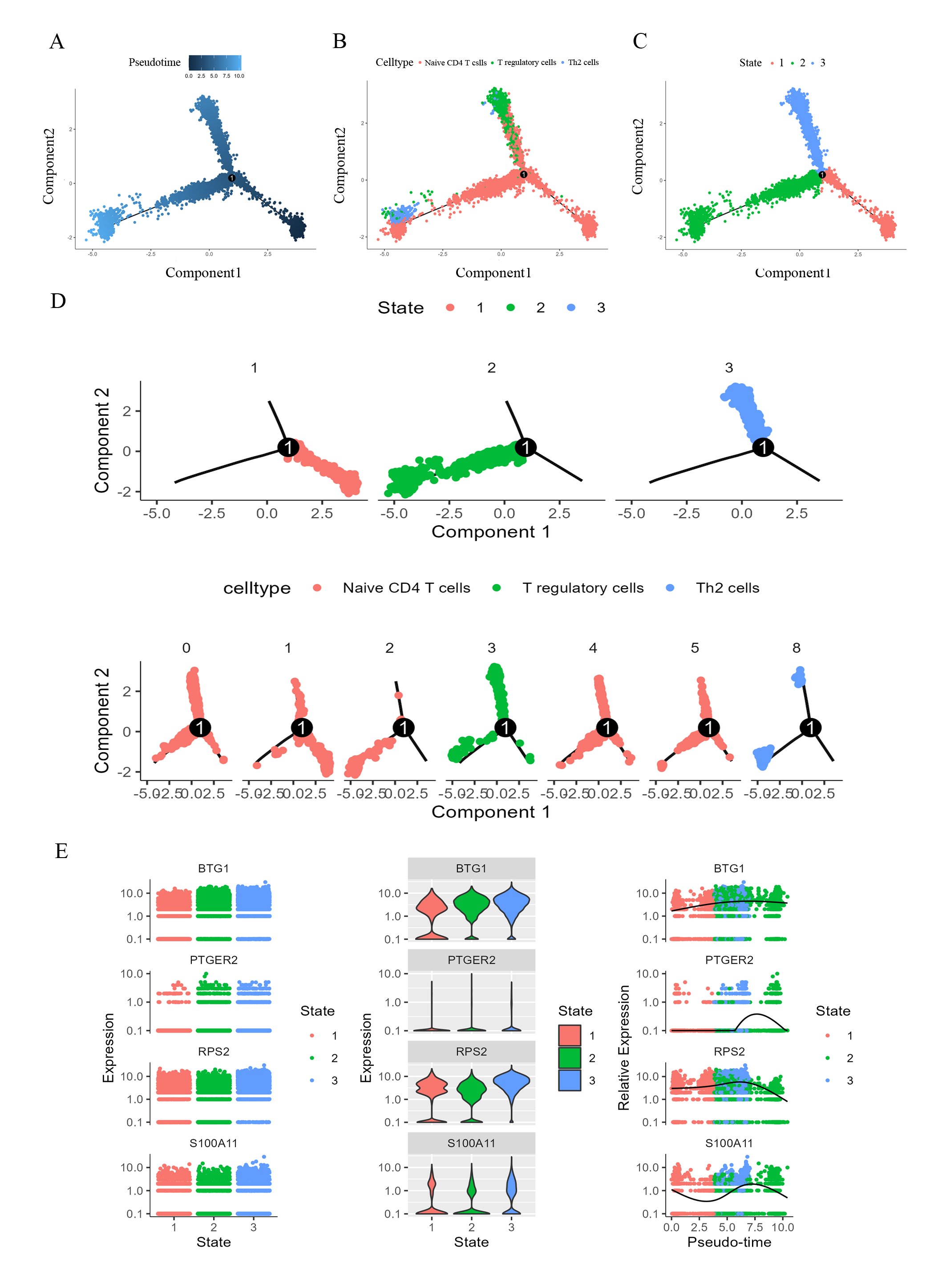


Supplement Figure 1 Detailed cell trajectories of CD4+T cells. (A) Label colors according to separate clusters. The darker the color, the closer it is to the root. (B,C) The results of pseudo time analysis show trajectory differentiation maps for three types of cells. (D) Displaying three cell subpopulations separately on each fate branch provides a clearer picture of the trajectory differentiation of cell types. (E) Select specific marker genes (BTG1, PTGER2, RPS2, S100A11) to fall on the entire cell trajectory, and observe the expression changes of each gene in the trajectory.
